# Evaluating ATP testing for distribution system monitoring: comparison to HPC, impact of chlorine quenching, and hold time dependency

**DOI:** 10.1186/s13036-024-00446-z

**Published:** 2024-11-05

**Authors:** William S. Chen, Leili Abkar, Madjid Mohseni

**Affiliations:** Department of Chemical and Biological Engineering, University of British Columbia, 2360 East Mall, Vancouver, Canada

**Keywords:** Utility management, Water quality, Heterotrophic plate count, Adenosine triphosphate, Distribution network, Microbial activity

## Abstract

Adenosine triphosphate (ATP) assays have a faster turnaround time and higher sensitivity than traditional cultivation methods for microbial monitoring. Challenges implementing ATP testing include incompatibility with chlorine quenching agents and hold time sensitivity, which are not well-studied. Chlorinated distribution system samples were collected from two Canadian utilities, Metro Vancouver (*n* = 40 samples) and Halifax Water (*n* = 283). No significant correlations were observed between heterotrophic cell count (HPC) and cellular ATP, suggesting these do not correlate well in waters with low biological activity (median HPC < 2 CFU/mL). However, interpretation of HPC and cATP results (based on the HPC guideline of 100 CFU/mL and cATP of 10 pg/mL) yielded the same conclusion for 95% of samples, suggesting a potential decision-making framework to replace HPC with cATP. Moreover, cATP correlates better with free chlorine (*p* < 0.04) compared with HPC for one of the studied systems. Importantly, adding chlorine quench (10% sodium thiosulfate) did not produce significantly different cATP results, nor did analyzing at various hold times of 4-, 6-, and 24-h. This study supports the integration of ATP testing into existing sampling procedures for water utilities, as a sensitive, fast, and reliable monitoring method.

**Graphical Abstract:**

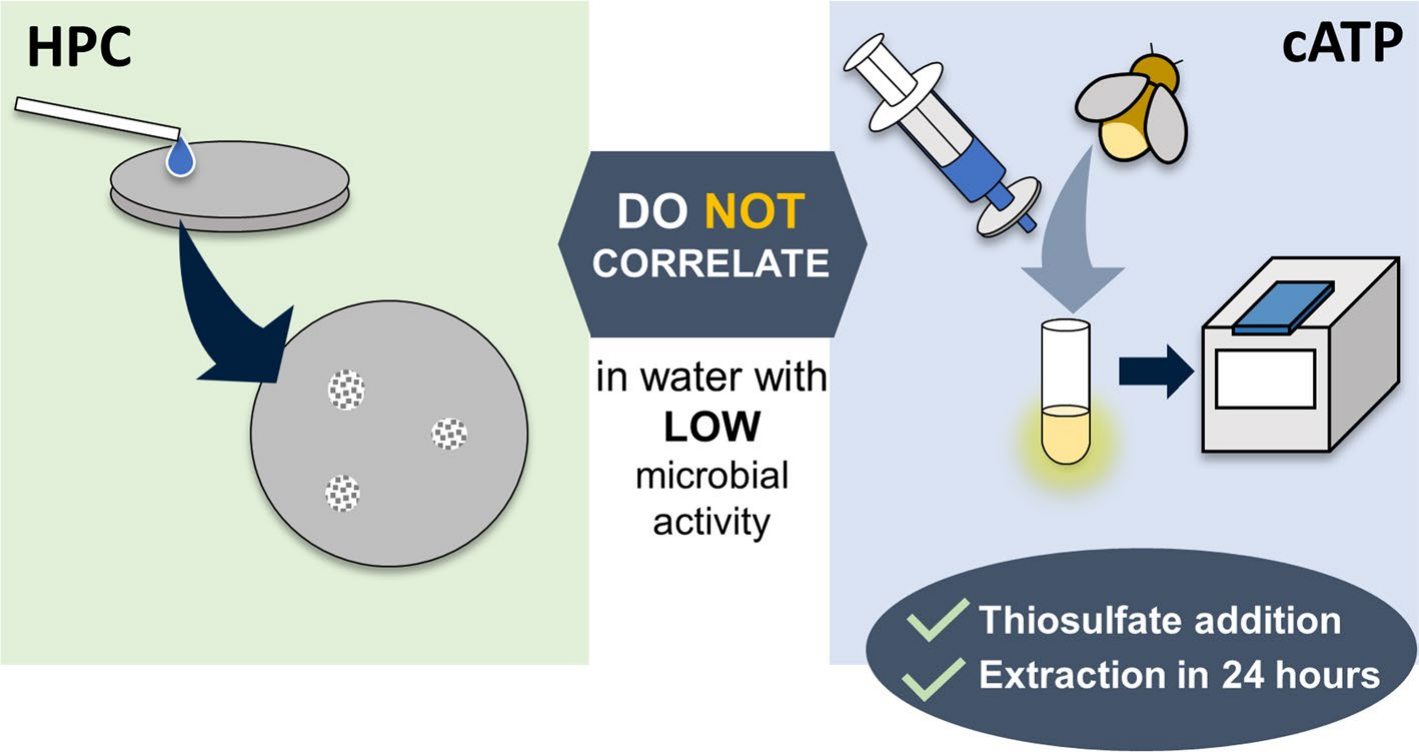

**Supplementary Information:**

The online version contains supplementary material available at 10.1186/s13036-024-00446-z.

## Introduction

Monitoring biological stability in potable water distribution systems is vital for ensuring consumer health and safety [17]. As water travels through the distribution system, the quality may deteriorate due to loss of disinfectant residuals (e.g., chlorine, chloramine), nitrification, biofilm detachments, corrosion, and when a component of the distribution system is being taken out for repair or replacement [5]. Standard methods (e.g., Standard Method 9221, 9222, 9223) are based on cultivation techniques, such as total coliform enumeration, *Escherichia coli (E.coli)* enumeration, and heterotrophic plate count (HPC). These cultivation methods are well-established and have been used as microbiological indicators to monitor bacterial regrowth in the distribution system. Factors promoting regrowth include operational/design conditions such as flow stagnation or insufficient disinfectant residuals, as well as environmental conditions like increased temperature and nutrient availability in the source water; these can result in increased biological activities which are measurable by HPC, *E.coli*, and total coliform results [2, 18]. However, a major disadvantage of these cultivation methods is the long turnaround time due to the 3- to 5-day incubation period. This can delay the release of infrastructure for service, whether for new installs, repairs, or maintenance, leading to increased construction time and costs, lost production, and negative impacts on users and communities particularly if drinking water advisories are prolonged [35]. Other concerns of slow turnaround time include missing transient or short-term peak contamination events and delayed implementation of corrective actions, both of which can potentially expose users to risks.

Another limitation is that these methods target a specific species or group of microorganisms (e.g., *E. coli* and total coliform), which are not fully representative of the bigger picture of microbial community structure. Further, HPC captures a small fraction of bacteria: typically only 0.01% of bacteria found in water are heterotrophic, of which only 1% are culturable [11]. HPC has been found to detect < 1% of total cell counts as determined by microscopy or flow cytometry [37]. As well, the small sample volume utilized in HPC (typically ≤ 1 mL) makes the method more sensitive to heterogeneity in the water and limits the ability of this method to accurately represent the water tested.

Adenosine triphosphate (ATP) measurement is increasingly being used for monitoring microbiological activity in water distribution systems [17]. It is referred to as the “energy currency” in viable cells and can be used independently for viability assessment of microbial cells [16]. Commercially available kits utilize an enzyme (luciferase, also found in fireflies) that reacts with ATP molecules to produce light, and the resulting luminescence is measured and compared against a standard to determine ATP concentration [22]. ATP testing captures both culturable and non-culturable cells that include nitrifiers, sulphate reducers, and eukaryotes [10, 40]. The test will also measure ATP in cells that are still alive but unable to reproduce, which could arguably lead to underestimating the extent of disinfection processes [40]. However, this is balanced against the fact that ATP tests can also determine the impact of disinfectants on a broader range of microorganisms than HPC [40]. One advantage of ATP is the quick turnaround time, with results being generated in seconds to minutes [38]. This can be particularly advantageous for time-sensitive applications, such as monitoring water safety when returning infrastructure to service e.g., after a line break or new installation [35]. As well, cellular ATP (cATP) testing kits, which typically capture cells on a filter for analysis, test a much larger volume of water (50–100 mL) than HPC, thus reducing the impact of sample heterogeneity on test results. It is noteworthy that both HPC and ATP are general indicators of biological activity and their predictive capabilities for specific drinking-water associated pathogens are limited [36].

Numerous studies have been performed to evaluate ATP and HPC correlation (Table 1) and a wide range of results have been reported (Pearson *R* or Spearman *R*_*s*_ from -0.13 to 0.93). Biologically active waters were associated with stronger correlations: high correlation coefficients (*R* or *R*_*s*_ > 0.8) were found in studies using waters with HPC values up to 10^5^–10^6^ CFU/mL [8, 10], whereas weak correlations (*R* or *R*_*s*_ < 0.4) all involved waters with HPC values no greater than 650 CFU/mL [4, 16, 19, 27, 31]. Poor correlation of HPC and ATP in low-activity waters could be attributed to the higher sensitivity of ATP assay, as microbial levels may be below the detection limit for HPC but still detectable by ATP testing; this discrepancy in sensitivity would thus contribute to a poor correlation between the two parameters. An insufficient upper limit for HPC may also have contributed to a weak correlation in some studies; for instance, Hammes et al. [16] tested samples with relatively high ATP of up to 10^6^ pg/mL (10^9^ microbial equivalents [ME] per mL, based on 0.001 pg ATP per cell [22]) but an HPC upper limit of only 300 CFU/mL and reported one of the lowest correlations (*R* = 0.06). Several studies also compared ATP and HPC against other monitoring parameters, such as intact cell count by flow cytometry (FCM) and residual chlorine, generally finding ATP to correlate better than HPC to these parameters [19, 27]. For instance, Kennedy et al. [19] determined *R*_*s*_ (Spearman) = -0.77 for ATP and free chlorine versus *R*_*s*_ = -0.22 for HPC and free chlorine in a chlorinated distribution system (*n* = 21). Similarly, Nescerecka et al. [27] determined *R*^2^ = 0.77 (*n* = 49) for intracellular ATP and FCM intact cell count, compared to *R*^2^ = 0.18 (*n* = 38) for HPC versus FCM intact cell count.

**Table 1:** Summary of literature review on HPC and ATP comparison studies involving drinking water. ATP converted to ME/mL based on 1 pg/mL = 1000 ME/mL (LuminUltra). PCA = plate count agar, YEA = yeast extract agar, R2A = Reasoner’s 2 agar. *R*: Pearson coefficient (linear), *R*_*s*_: Spearman coefficient (ranked)

Country	Water Type	Location	ATP system	HPC media	Correlation	ATP Range (ME/mL)	HPC Range (CFU/mL)	Sodium Thiosulfate	Reference
USA	Chlorinated^a^ drinking water	Usage point	Luminultra QGA	R2A	*R* = 0.90 *n* = 106	40–1 × 10^5^	2–3 × 10^5^	Yes	[10]
	Chlorinated drinking water	Distribution system	ProMega BacTiter-Glo	- ^b^	*R*_*s*_ = 0.13 *n* = 21	10–6000	0.01–2.3	Yes	[19]
	Chloraminated drinking water	Distribution system	ProMega BacTiter-Glo	- ^b^	*R*_*s*_ = 0.37 *n* = 61	10–15000	0.01–24	Yes	[19]
	Drinking water	Not specified	New Horizons Diagnostics	R2A	*R* = 0.93 n≈50	- ^c^	5–1 × 10^6^	No	[8]
Canada	Chlorinated drinking water	Usage point	GL-Biocontrol Dendriag SW	R2A	*R*_*s*_ = 0.43 *n* = 150	0–35000^d^	1–1 × 10^4 d^	Yes	[12]
Switzerland	Non-chlorinated^a^ drinking water	Usage point	Promega BacTiter-Glo (total ATP)	PCA	*R* = 0.56^e^ *n* = 200	1000–48000	1–16000	No	[34]
	Non-chlorinated drinking water	Usage point, bottled water	ProMega BacTiter-Glo (total ATP)	R2A	*R* = 0.51^e^ *n* = 27	15000–55000	50–9000	No	[4]
				PCA	*R* = 0.17^e^ *n* = 27	15000–55000	1–630	No	[4]
	Surface water, groundwater, non-chlorinated drinking water, wastewater	Source, usage point, effluent (wastewater)	ProMega BacTiter-Glo	R2A	*R* = 0.06^e^ *n* = 102	250–1 × 10^6^	30–300^f^	No	[16]
France	Chlorinated drinking water	Distribution system	New Horizon Diagnostics	R2A	*R* = 0.60 *n* = 64	2–4000	5–10000	No	[9]
Netherlands	Non-chlorinated^g^ drinking water	Usage point, effluent	Celsis	YEA	*R* = -0.13 *n* = 175	1000–4500	1–140	No	[31]
	Non-chlorinated drinking water	Effluent, usage point	Celsis (total ATP)	R2A	*R* = 0.44^e^ *n* = 56	500–13000	1–4500	No	[37]
Latvia	Chlorinated drinking water	Distribution system	Promega BacTiter-Glo	PCA	*R* = 0.33^e^ *n* = 38	0–4.6 × 10^5^	1–220	No	[27]
Various	Drinking water	Not specified	New Horizons Diagnostics	R2A	*R* = 0.86 *n* = 120	- ^c^	1–3 × 10^5^	No	[8]

^a^Not specified by authors

^b^IDEXX Quanti-Tray for HPC (does not use culture media)

^c^ATP results only reported in RLU

^d^Range of HPC and ATP is of the full dataset (*n* = 375 and *n* = 475, respectively), of which a subset (*n* = 150) is used for HPC-cATP correlation

^e^Authors reported result in *R*^2^

^f^Detection limit

^g^Treatment system uses chlorine dioxide for disinfection, but no residual disinfectant is maintained in the distribution system

While ATP has shown promising results and application in determining the biological activity in different water sectors, a potential operational challenge is its incompatibility with chlorine quenching agents, which are used for sample preservation in other bacteriological tests. The use of quenching agents such as sodium thiosulfate is not recommended by the test kit manufacturer [21]. At the same time, implementing different sample handling procedures can be prohibitively cumbersome for utility managers. Commercial ATP kits are designed for field use to allow analysis immediately after collection, as ATP begins changing immediately under non-native conditions. However, having sampling staff perform ATP extractions in the field may not be feasible. Test kit manufacturers state ATP extraction within 24 h is acceptable if samples are kept below 5 °C, but the extent of variation under these conditions is not well-studied.

The present study aimed to assess the potential correlation of ATP and HPC in chlorinated distribution systems to understand the accuracy and reliability of replacing HPC with ATP testing. It also investigated its correlations to other monitoring parameters, namely turbidity and free chlorine. For the first time, this study further evaluated the critical operational considerations, such as the impact of the quenching agent addition (i.e., sodium thiosulfate) and variable hold times on ATP results.

## Materials and methods

### Description of study sites

#### Metro Vancouver

Greater Vancouver Water District (GVWD) is an entity of Metro Vancouver responsible for treating and supplying potable water to 2.7 million residents [26] in the jurisdictions including and surrounding Vancouver, British Columbia [39]. Water is sourced from three lakes collecting rainfall and snowmelt from surrounding mountains and is characterized by: average pH 6.3–6.5, dissolved organic carbon (DOC) 1.6–1.8 mg/L, turbidity 0.5–1.7 NTU, and total residue (solids) 12–16 mg/L [25]. Source water is treated at two facilities before being delivered to member jurisdictions via a water transmission network 520 km in length (the scope of the present study), along which the transmission mains branch out to various localized distribution piping networks thousands of kilometers in length [24]. Sodium hypochlorite (bleach) is added to treated water at both treatment facilities and maintained by eight secondary chlorination stations throughout the transmission network, with target chlorine residuals ranging from 0.8 to 1.5 mg/L at the chlorination station outlet [25].

#### Halifax Water

Halifax Water provides potable water to 360,000 residents in the city of Halifax (Nova Scotia, Canada) and surrounding areas through nine water treatment systems and a distribution network approximately 1570 km in length [13, 14]. Various treatment technologies are employed in the nine systems, including disinfection by either chlorine gas or sodium hypochlorite injection. Residual chlorine is maintained above 0.2 mg/L in the distribution system using sodium hypochlorite [15].

### Experimental design

From the Halifax Water system, samples were collected from 11 sampling sites along the distribution system (prior to customer taps) from January to November 2021, for a total of *n* = 283 samples. Halifax Water provided HPC, cATP, and free chlorine analysis results to the authors. From the Metro Vancouver system, samples were collected from the inlet and outlet of the two treatment facilities and 10 monitoring locations along the transmission system (i.e., main trunk serving multiple municipal distribution networks) from June to July 2022. This resulted in *n* = 40 samples which were analyzed for HPC, cATP, turbidity, and free chlorine. Together with the Halifax Water results, these data were used to study correlation between the water quality parameters.

Additional samples were collected, from the Metro Vancouver system only, to further study the robustness of cATP analysis. This portion of the study consisted of the following:

#### Establishing baseline of cATP variance

Variance of cATP was assessed by analysis in triplicates of samples collected from four sampling locations, selected based on historical high/low HPC/free chlorine concentrations to capture the full range of HPC and free chlorine encountered in the treatment and transmission systems (detailed in Table S1). The coefficient of variance for each triplicate set was calculated as the standard deviation divided by the mean. A total of *n* = 26 sets of triplicates were collected from the four sampling locations from June to July 2022, which includes samples with and without thiosulfate addition extracted at 4, 6, and 24 h after collection.

#### Impact of quenching agent

To assess the impact of a quenching agent, sodium thiosulfate (sodium thiosulfate pentahydrate, 99.5–101%, Anachemia [VWR]), on cATP results, select samples (*n* = 7) were additionally collected in bottles without sodium thiosulfate (unquenched) for comparison against samples collected in standard bottles pre-charged with 10% sodium thiosulfate (quenched).

#### Impact of hold time

To assess the impact of hold time, cATP extraction was performed at multiple specific time points for select samples with thiosulfate (*n* = 13) and without thiosulfate addition (*n* = 7), at 6 and 24 h after collection, as well as at 4 h after collection where possible (transit time for samples to reach laboratory ranged between 4 to 6 h after collection). Samples were stored below 5 °C between extractions.

### Sample collection and water quality analysis

#### Metro Vancouver

Sample lines were lightly flushed for 5 min, then samples were collected in 250 mL plastic Nalgene bottles, pre-charged with approximately 0.2 mL of 10% sodium thiosulfate, and transported to the laboratory in coolers with cold packs within 6 h after collection. Samples were stored at 4 °C.

Temperature and free chlorine were measured during sample collection using a multiparameter probe (Hach® Pocket Pro Tester) and field kits, respectively. Turbidity was measured in the laboratory using a turbidimeter (Hach® TL2300).

HPC was conducted using the spread plate method using R2A media to inoculate 0.5 mL of sampled water. The plates were incubated at 28 °C over 5 days. Analysis of cATP was performed using a commercial reagent kit (LuminUltra® QuenchGone™ Aqueous [QGA]) and a luminometer (LuminUltra® PhotonMaster). Per manufacturer instructions, within 24 h of collection, samples were filtered to capture cells and subsequently lysed to extract cellular ATP (any extracellular ATP was discarded during filtration). Lysates were stored at 5 °C and analyzed within 7 days of extraction.

#### Halifax Water

After light flushing, samples for HPC analysis were collected in sealed, sterile bottles precharged with 10% sodium thiosulfate supplied by a third-party laboratory. Samples for cATP were collected directly into 50 mL sterile syringes provided by the manufacturer for field extraction. HPC samples and cATP lysates were transported to the lab in coolers with ice or ice packs. Free chlorine was analyzed at time of collection using field kits.

HPC was conducted by a third-party laboratory using the spread plate method with R2A media: 1 mL of sampled water was inoculated and incubated at 35 ± 0.5 °C for 48 ± 3 h. Testing for cATP was performed using the same commercial kits and luminometer as the Metro Vancouver study (LuminUltra® QGA and PhotonMaster). As discussed above, cATP samples were filtered and extracted in the field immediately after collection. Upon receipt at the lab, lysates were refrigerated and stored for up to one week prior to analysis, as per manufacturer instructions.

### Statistical analysis

The correlation of sampled parameters was determined with both Pearson correlation coefficient *R* (least squares method), and Spearman ranked correlation coefficient *R*_*s*_. The significance of the correlation was determined by the t-test. The normality of regression residuals was assessed by the Shapiro-Wilks test. Results below the lower detection limit were taken as half the detection limit (1 CFU/mL Halifax Water, 2 CFU/mL Metro Vancouver), and results above the maximum upper limit were taken as equal to the upper limit (250 CFU/mL Halifax Water; no results for Metro Vancouver exceeded the upper limit of 5700 CFU/mL).

The significance of the difference in cATP results for samples with and without thiosulfate (paired dataset) was assessed at three different extraction times: 4, 6, and 24 h after sample collection. Paired t-test was used for paired data sets with normal distribution, as confirmed by the Shapiro-Wilks test. Otherwise, Wilcoxon signed-rank test was performed. Comparison of percent differences in cATP results (e.g., between different extraction times) versus baseline variance was conducted using a two-sample t-test without assuming equal standard deviation, i.e., Welch’s test. Statistical calculations were performed in R and regression residual plots were produced using Excel.

## Results and discussion

### Studied waters exhibit low microbial activity

A summary of parameters in samples collected in the Metro Vancouver and Halifax Water systems is presented in Table 2:

**Table 2:** Characteristics of water samples collected from Metro Vancouver and Halifax Water transmission/distribution systems. Median results are shown, and the interquartile range (IQR) is represented in parenthesis (no value indicates an IQR of 0). Turbidity results from Halifax Water are not available

Parameter	**Halifax Water**	**Metro Vancouver**	
	Treated Water *n* = 283	Treated Water *n* = 32	Raw Inlet *n* = 8
HPC (CFU/mL)	< 1 (< 1–1)	< 2 (–)	180 (91–358)
cATP (pg/mL)	0.20 (0.07–0.49)	0.20 (0.11–0.33)	23.0 (8.2–38.0)
Free chlorine (mg/L)	0.57 (0.45–0.77)	0.82 (0.71–0.93)	N/A
Turbidity (NTU)	N/A	0.23 (0.17–0.28)	0.31 (0.27–0.35)

Treated water samples are comparable between the two sites and characterized by low microbial activity. Median HPC are below detection limits for both systems, with 78% of Metro Vancouver treated water samples (*n* = 25 out of 32) and 67% of Halifax Water samples (*n* = 190 out of 283) being non-detectable for HPC. Maximum value of HPC is 14 CFU/mL for Metro Vancouver, while 2% (*n* = 7) of Halifax Water samples exceed the relatively low upper limit of 250 CFU/mL. In comparison, studies showing high HPC-ATP correlation such as Duda et al. [10] reported HPC up to 10^5^ CFU/mL, which is far greater than even Metro Vancouver raw inlet results (maximum HPC of 400 CFU/mL).

Median cATP in treated water samples for both study sites is 0.20 pg/mL, with a maximum of 2.5 pg/mL for Metro Vancouver and 7.9 pg/mL for Halifax Water. All treated water samples are therefore below the recommended limit of 10 pg/mL for corrective action [23]. cATP demonstrates a higher sensitivity than HPC, as expected: only 12% of Halifax Water samples (*n* = 34) are non-detectable for cATP, and none of the Metro Vancouver samples are non-detectable.

### Poor correlation of cATP and HPC in waters with low microbial activity

**Fig. 1:**
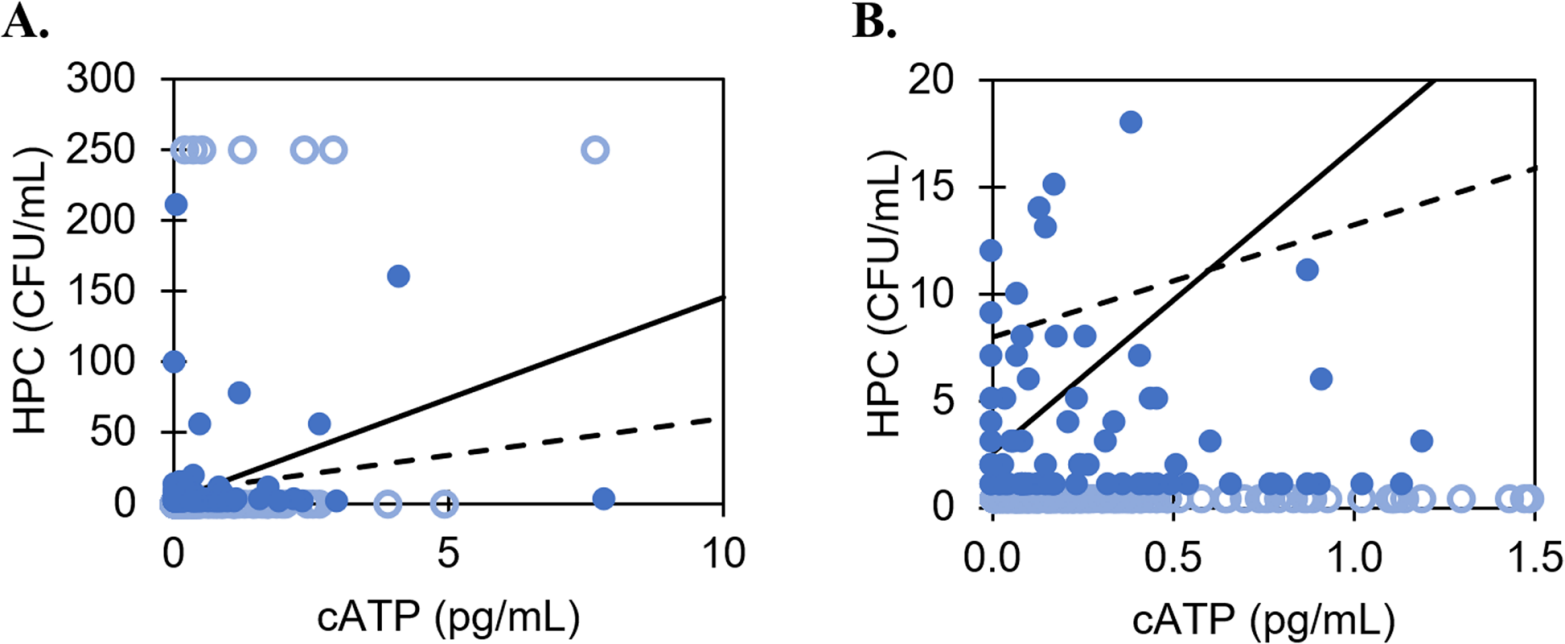
**A** HPC versus cATP for Halifax Water system samples collected from January to November 2021. **B** zooms into data near the origin for better visibility. Solid circles = samples with HPC values between lower and upper limit (*n* = 86); hollow circles = samples with HPC values below/above the lower/upper limits (*n* = 197); solid line = regression line of all samples (*R* = 0.31, *p* << 0.001, *n* = 283); dashed line = regression line of samples between HPC lower and upper limits (*R* = 0.18, *p* = 0.09, *n* = 86)

**Fig. 2:**
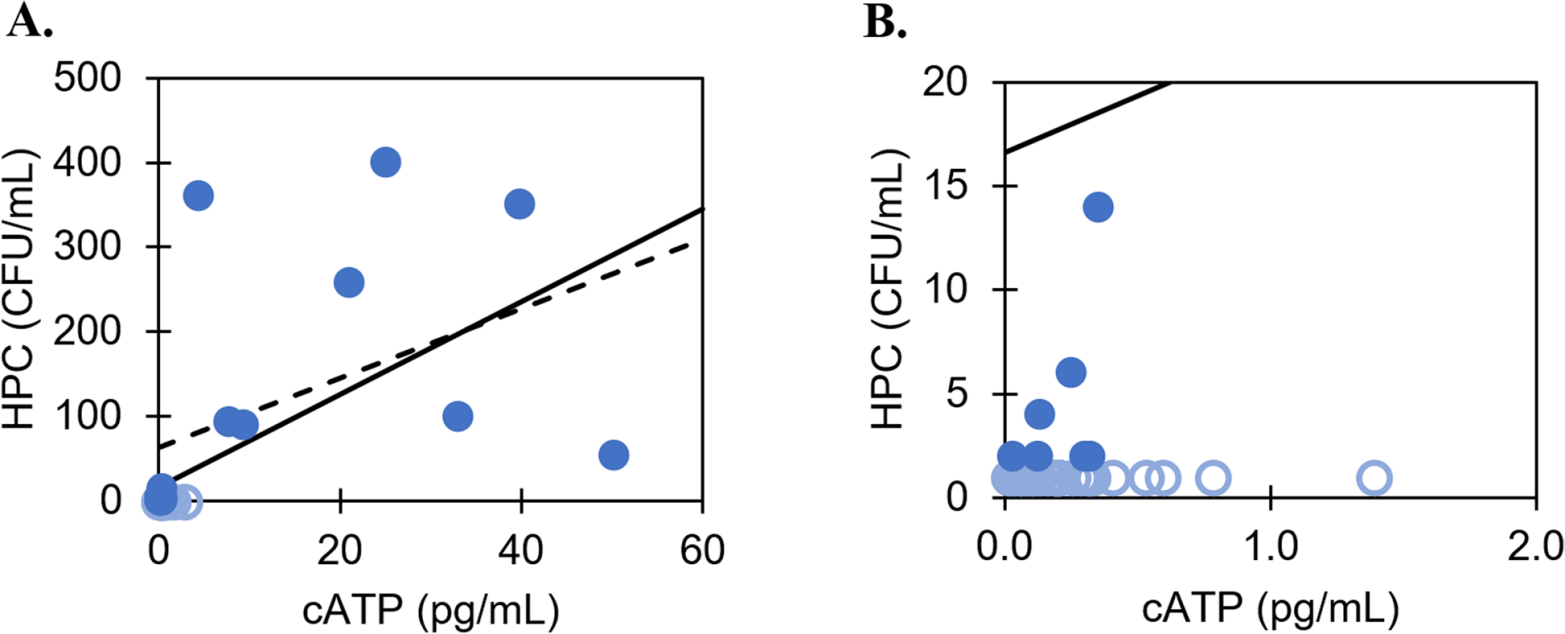
**A** HPC versus cATP for samples collected from Metro Vancouver inlet water and along the transmission system from June to July 2022. **B** zooms into data near the origin for better visibility. Solid circle = samples with HPC above detection limit (*n* = 15). Hollow circle = samples with HPC below the detection limit (*n* = 25). Solid line = least-squares regression line of all samples (*R* = 0.61, *p* << 0.001, *n* = 40). Dashed line = least-squares regression of samples above detection limits only (*R* = 0.46, *p* = 0.08, *n* = 15)

HPC and cATP results for the Halifax Water and Metro Vancouver studies are presented in Figs. 1 and 2, respectively.

While Pearson *R* coefficients are shown to facilitate comparison with other studies, the data do not, in fact, satisfy the assumptions for simple linear regression. Namely, both Halifax Water and Metro Vancouver datasets yielded significant non-normal distributions of residuals, skewed left (*p* << 0.001; residual plots, histograms, and Q-Q plots are shown in Figure S1 and Figure S2). One main contributor to the asymmetrical distribution appears to be the disproportionate number of low HPC samples (< 2 CFU/mL), combined with the fact that these low-range HPC samples correspond to a wide range of cATP results (0 to 3 pg/mL). This results in a significant number of negative residuals in the low HPC/cATP range (i.e., left-skewed), which is not improved even after excluding samples below and above the HPC detection (*p* << 0.001).

Spearman *R*_*s*_ coefficient is therefore considered a more appropriate descriptor for these datasets, summarized in Table 3 for all studied parameters (equivalent correlation matrix for Pearson *R* coefficients provided in Table S2, for reference):

**Table 3:**
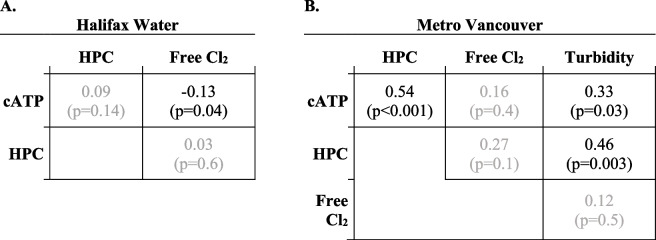
Spearman R_*s*_ calculated for (A) Halifax Water (*n* = 283) and (B) Metro Vancouver (*n* = 40). Insignificant correlations (*p* > 0.05) are shown in grey. Correlations involving free chlorine for Metro Vancouver are limited to *n* = 32 treated water samples as free chlorine was not analyzed for *n* = 8 raw inlet samples

No significant relationship between HPC and cATP is observed for Halifax Water (*p* > 0.05). In contrast, a significant relationship of moderate strength is observed for Metro Vancouver (*R*_*s*_ = 0.55, *p* < 0.001). This aligns with studies testing HPC waters of a similar range: Nescerecka et al. [27] found *R*_*s*_ = 0.33 when testing chlorinated water distribution samples with HPC up to 220 CFU/mL. The likely reason for the discrepancy between Halifax Water and Metro Vancouver is the inclusion of raw source water in the Metro Vancouver dataset: the relatively higher HPC and cATP values of the source water samples allow for a relationship to be determined between these two parameters. Excluding these raw water samples (*n* = 8) eliminates any significant relationship between HPC and cATP (*p* = 0.9, *n* = 32; detailed results provided in Table S3).

Therefore, the present study supports the observation that low HPC waters, in the ranges typically found in chlorinated potable distribution systems, are unlikely to produce strong correlations between HPC and ATP. This also can be due to the fact that ATP is not sufficiently accurate in aquatic environments with low microbial concentrations [16]. In comparison, studies finding a high correlation, such as *R* = 0.90 by Duda et al. [10] involved chlorinated waters collected at point-of-use with a mean HPC of 537 CFU/mL (reported as log_10_), which are orders of magnitude higher than the present study median HPC that is below detection limits (< 1–2 CFU/mL).

A limitation of ATP analysis, which can contribute to discrepancies with HPC, is that not all cells may be successfully lysed during sample preparation. Interestingly, while Gram-positive bacteria have a thicker peptidoglycan layer that provides structural support and resistance to chemical agents, Turner et al. [36] determined that it is in fact Gram-negative bacteria which experience incomplete cell lysis, thus resulting in underestimation of ATP levels. Additionally, the presence of an outer membrane in Gram-negative bacteria contributes to their lower surface charge, ranging from -80 to -140 mC m^−2^, compared to the higher surface charge of -350 to -450 mC m^−2^ in Gram-positive bacteria [7]. This difference in bioelectrical environments affects the efficiency of ATP extraction and measurement. The outer membrane's complex structure in Gram-negative bacteria hinders processes like electron transfer, which are crucial for accurate ATP measurement, leading to potential inaccuracies [28]. Conversely, ATP measurement in Gram-positive bacteria is typically more straightforward, with a more linear relationship between microbial suspension density and ATP concentration [33]. Therefore, the differences in cell wall structure, bioelectrical properties, and the methodologies used significantly influence the accuracy of ATP measurements across these bacterial groups. Gram-negative bacteria can comprise a significant portion of the microbial population in surface waters, making up as much as 90% of isolates studied in Lake Ontario and Lake Superior [3]. Moreover, the percentage of Gram-negative bacteria can also shift significantly from source to point-of-use: Pepper et al. [30] found Gram-negative bacteria comprised 76% of the studied source river water but only 12% in the chlorinated distribution system and 0.2% at the tap. This may explain the poor correlation between HPC and ATP in water samples, even in waters with higher biological activity.

Additionally, some of the concerning pathogenic heterotrophic bacteria that are Gram-negative are total coliforms, *E. coli, Shigella, Pseudomonas aeruginosa, Legionella pneumophila, Aeromonas hydrophila, Klebsiella pneumoniae, Salmonella enterica* [6]*.* Therefore, considering the prevalence, variability, and pathogenic concern of Gram-negative bacteria, it is advisable to complement ATP testing with species-specific tests, particularly for systems that utilize source waters of pathogenic concern. Alternatively, ATP detection limits for the mentioned pathogenic species can also be established in pure culture form. For instance, Turner et al. [36] reported that the detection limit of *E*. *coli* and *Staphylococcus aureus* was 10^4^ and 10^2^ organisms, respectively.

### Interpretation of HPC and cATP results can yield consistent conclusions

**Fig. 3:**
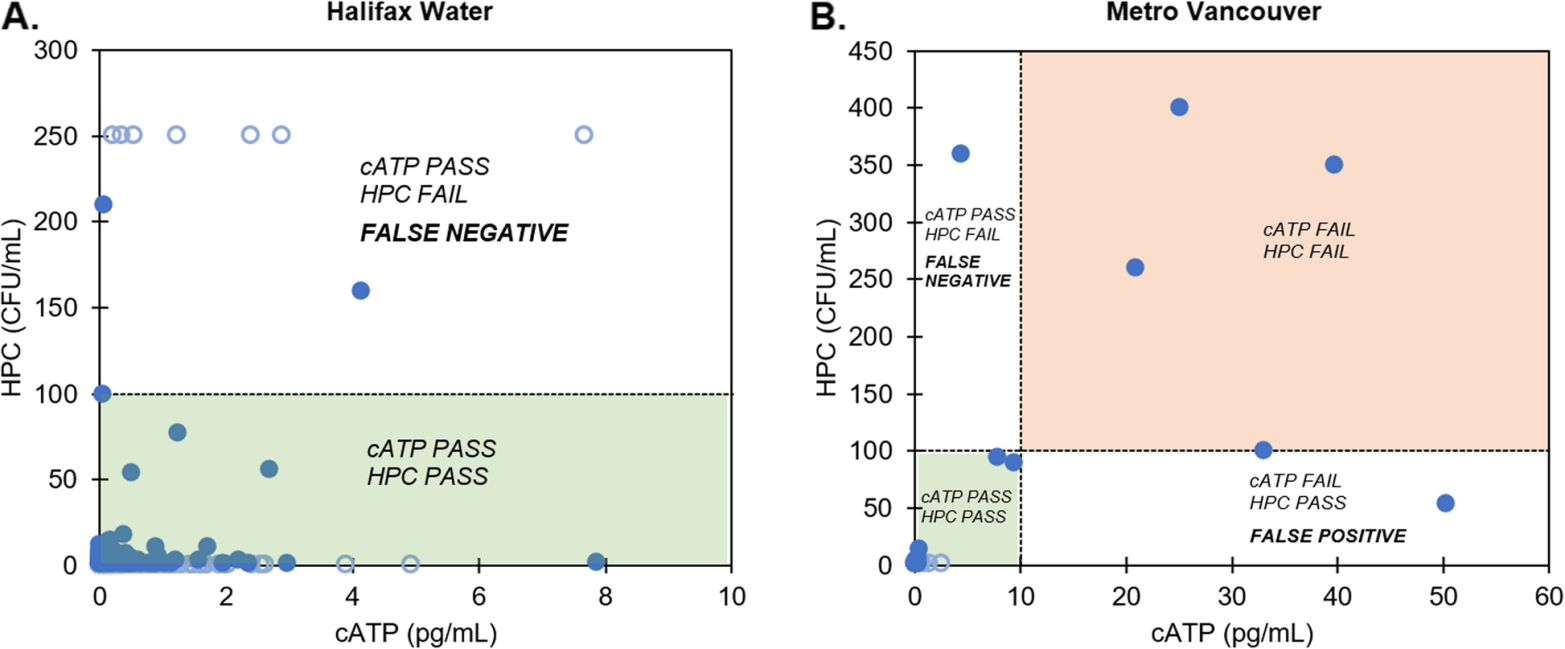
HPC and cATP data for Halifax Water (**A**) and Metro Vancouver (**B**), previously presented in Figs. 1 and 2 respectively, shown again here with pass/fail criteria of HPC = 100 CFU/mL and cATP = 10 pg/mL. Solid circles = HPC within detection limit, hollow circles = HPC outside of detection limit

Despite weak correlation between cATP and HPC, it is worth noting that using guideline ranges to interpret cATP and HPC data can lead to highly consistent conclusions. To illustrate, HPC results ≥ 100 CFU/mL can be considered indicative of a need for investigation, based on the drinking water standard limit of 100 CFU/mL employed in jurisdictions like Germany, The Netherlands, and Japan [32] (in comparison, the US EPA guideline for HPC of 500 CFU/mL [18] is too high to be relevant for the studied waters, none of which exceeded this value). Similarly, cATP results ≥ 10 pg/mL has been suggested by the test kit manufacturer as being indicative of a need for corrective action [23]. Application of this pass/fail criteria to the data set can be visualized as follows:

For the Halifax Water study, 96% of the samples (*n* = 273) are in agreement between cATP and HPC, in that they pass both cATP criterion (< 10 pg/mL) and HPC criterion (< 100 CFU/mL; Fig. 3A, bottom shaded region). The remaining 4% (*n* = 10) pass cATP (< 10 pg/mL) but fail HPC (≥ 100 CFU/mL; Fig. 3A, top unshaded region); these can be described as false negatives if cATP is treated as the predicting parameter, i.e., cATP “wrongly” identifies a sample as exhibiting low microbial activity when HPC is in fact elevated. None of the samples from Halifax Water exceed the cATP fail criterion of 10 pg/mL.

For the Metro Vancouver study, 95% of the samples (*n* = 38) agree between HPC and cATP; samples either pass both criteria (*n* = 34, Fig. 3B, bottom left shaded region), or fail both criteria (*n* = 4, Fig. 3B, top right shaded). One false negative of low cATP and high HPC is observed (Fig. 3B, top left unshaded), along with one false positive of high cATP and low HPC (Fig. 3B, bottom right unshaded). These results are summarized in Table 4:

**Table 4:** Analysis of error probability, based on criteria of cATP ≥ 10 pg/mL and HPC ≥ 100 CFU/mL as indicative of the need for corrective action. False positive: cATP indicates corrective action (≥ 10 pg/mL), but HPC does not (< 100 CFU/mL). False negative: cATP does not indicate corrective action (< 10 pg/mL) but HPC does (≥ 100 CFU/mL). Correct: cATP and HPC results are consistent (both above or both below 10 pg/mL and 100 CFU/mL, respectively)

cATP Interpretation Compared to HPC	Halifax Water *n* = 283	Metro Vancouver *n* = 40
Correct	96% (*n* = 273)	95% (*n* = 38)
False positive *High cATP, low HPC*	0%	2.5% (*n* = 1)
False negative *Low cATP, high HPC*	4% (*n* = 10)	2.5% (*n* = 1)

To be clear, this error analysis is only provided for illustrative purposes to demonstrate how cATP data may be used by utility managers to replace HPC. In practice, system-specific baselines and trends should be established for cATP, HPC and any other bacteriological monitoring technique (e.g., cell count using flowcytometry, gene copies count using q-PCR, bacterial relative and absolute abundance using microbiome analysis, etc.) over a sufficient test period, which is consistent with the recommended practice for how these bacteriological indicators are best used for utility monitoring [1, 17, 20].

### Correlating free chlorine and turbidity to HPC and cATP

Correlations between free chlorine, turbidity, HPC, and cATP for Metro Vancouver are summarized in Table 3. The Pearson coefficients for the same data are provided in Table S2. A weak but statistically significant negative correlation between free chlorine and cATP is observed for Halifax Water (*R*_*s*_ = -0.13, *p* = 0.04); the negative relationship is consistent with the disinfecting action of free chlorine. On the other hand, no significant relationship is observed between free chlorine and HPC for Halifax Water (*p* > 0.05). This is in agreement with Kennedy et al. [19] who reported a better correlation of cATP with free chlorine than HPC.

For Metro Vancouver, no correlation could be observed between free chlorine and cATP, nor between free chlorine and HPC. This disagreement with Halifax Water data could be attributed to the smaller sample size of the Metro Vancouver study, and it is possible that more samples collected over a longer period of time to capture a wider range of system conditions would have resulted in a significant correlation between free chlorine and cATP. It is also worth noting that raw inlet water samples (*n* = 8) were not analyzed for free chlorine and have been excluded from this analysis. If inlet samples are included with an assumed free chlorine of 0 mg/L, a significant correlation between cATP and free chlorine does emerge (*R*_*s*_ = -0.39, *p* = 0.01), though the same is also true for HPC and free chlorine (*R*_*s*_ = -0.44, *p* = 0.005). Notwithstanding, the present data only permits the tentative conclusion that cATP may correlate better than HPC to free chlorine, on the basis that one study determined significant correlation between cATP and free chlorine, whereas neither study found a correlation between HPC and free chlorine.

Turbidity data is only available from Metro Vancouver. Similar correlations between cATP and turbidity versus HPC and turbidity are observed (*R*_*s*_ = 0.33 and 0.46, respectively), i.e., neither appear to be obviously advantageous over the other. Furthermore, these correlations can be attributed to the inclusion of raw source water samples, without which no significant relationships (*p* > 0.05) are observed between turbidity and either HPC or cATP (Table S3). Therefore, the study results do not necessarily indicate that turbidity can be practically correlated to either parameter when sampling from chlorinated transmission/distribution systems.

### Sodium thiosulfate does not significantly affect cATP

As regulated microbial tests like for *E. coli* require samples to be chlorine-quenched, the impact of chlorine quenching on cATP assay was studied to assess whether thiosulfate-pre-charged sampling bottles can be used (i.e., to unify sample handling). As discussed in Methods Section "Impact of quenching agent", *n* = 7 pairs of samples with and without thiosulfate addition were collected and extracted at three time points, resulting in up to *n* = 6 pairs of comparative cATP results at each time point (not all samples could be extracted at all three time points) for a total of *n* = 15 data pairs (Table 5).

**Table 5:** cATP results for samples with and without thiosulfate addition, reported as median with interquartile range represented in parentheses. Percent difference (calculated as “no thiosulfate” minus “with thiosulfate”) reported as mean ± one standard deviation

**Time of extraction**	**cATP (pg/mL)**		**Percent difference**	
	**With Thiosulfate**	**No Thiosulfate**		
4 h	0.13 (0.13–39.98)	0.32 (0.29–23.70)	36 ± 77%	*n* = *3*
6 h	0.65 (0.17–29.95)	0.43 (0.23–17.13)	18 ± 123%	*n* = *6*
24 h	0.23 (0.12–7.12)	0.28 (0.19–12.08)	23 ± 89%	*n* = *6*

Median cATP results for samples with and without thiosulfate are comparable across the extraction times, with a mean percent difference of up to 36% (non-thiosulfate cATP higher than thiosulfate). Paired t-test and Wilcoxon signed rank test (the latter for non-normally distributed sample sets) conducted for thiosulfate versus non-thiosulfate results, at each extraction time all indicate a lack of significant difference (*p* > 0.4, detailed results in Table S4), suggesting that thiosulfate addition does not significantly impact cATP results at the conditions tested.

To further support the analysis of these results, the baseline variance of cATP is determined from the mean coefficient of variance for *n* = 26 sets of triplicates (detailed results provided in Table S5). The one-way ANOVA test confirms that the coefficients of variance evaluated under the various sampling conditions (Methods section "Establishing baseline of cATP variance") are not significantly different (*p* = 0.4). As such, all *n* = 26 triplicates are used to calculate a mean coefficient of variance of 35 ± 17%, which is consistent with literature values of 32 ± 16% [29]. Percent differences evaluated between thiosulfate versus non-thiosulfate (Table 5) are not significantly different from baseline variance (*p* > 0.7, Welch’s t-test; detailed results in Table S6), further supporting the conclusion that thiosulfate addition does not significantly impact cATP results.

### Extraction within 24 h is acceptable for cATP analysis

**Fig. 4:**
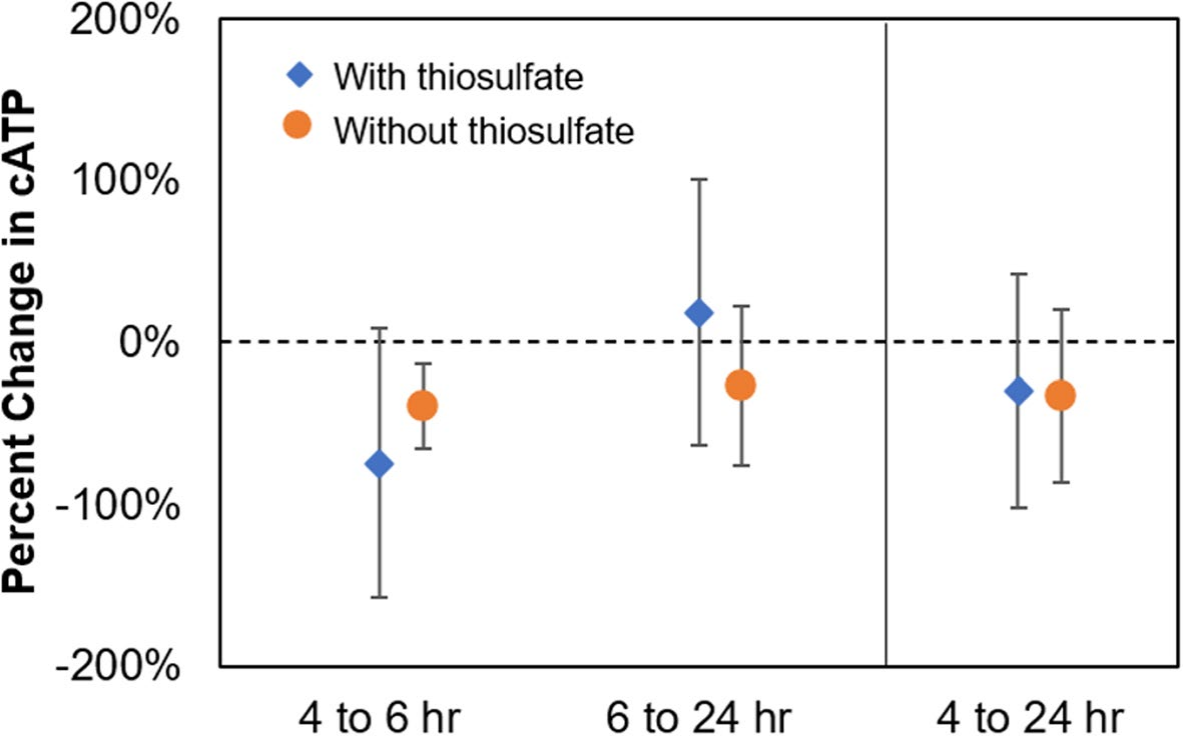
Percent change in cATP values between the extraction times studied (i.e., 4, 6, 24 h) for samples with and without sodium thiosulfate addition. Values shown are mean ± one standard deviation. Note that 4 to 24 h percent change is not necessarily the cumulative result of 4 to 6 h and 6 to 24 h because only select samples could be extracted at 4 h, with the result that different sample pairs are used to calculate each percent change (detailed breakdown provided in Table S7)

To assess the impact of sample extraction time on cATP results, samples were extracted at 4, 6 and 24 h after collection. Percent changes in cATP between different extraction times are presented in Fig. 4 (values reported in Table S7):

Samples without thiosulfate demonstrate a consistent decrease in cATP by 30 to 40% over 24 h, with an average standard deviation of 43%. Reduction in cATP would be consistent with the absence of chlorine quench by thiosulfate; chlorine in the samples is expected to accelerate cell death and reduce cATP over time.

Samples with thiosulfate show an inconsistent trend. A significant decrease (-75%) is observed over a relatively short time period of 4 to 6 h, followed by a small increase (+ 19%) from 6 to 24 h. Overall, from 4 to 24 h, cATP decreases by 30%, which is comparable to samples without thiosulfate (-34%). Notably, a larger standard deviation (average 79% over the three studied time-pairs) is observed than that for samples without thiosulfate (average 43%). The significant decrease from 4 to 6 h (-75%, *n* = 5 pairs) is difficult to explain, especially as the expected effect of thiosulfate addition would be improved preservation of cATP. It is worth noting that, of the five data pairs averaged in this calculation, two of the greatest changes (most negative) are observed for samples of relatively low cATP (0.13 pg/mL). That is, while a very large percent change is observed for these two sample pairs (-150%), the actual change in cATP for these samples is only 0.12 pg/mL, which is fairly small compared to the scale of cATP alert criterion (10 pg/mL). In other words, it is worth noting that the large 75% decrease from 4 to 6 h is the result of relatively small changes to already-low cATP values.

Despite these various apparent trends, none of the percent changes, whether for samples with or without thiosulfate, are statistically different than baseline variance (35 ± 17%). This is confirmed by Welch’s t-test yielding *p* > 0.3 at all conditions (statistical test conducted using absolute value of percent changes; detailed results in Table S8 and Table S9). In other words, fluctuations in cATP readings over the 24-h period can still be attributed to baseline variance, thus supporting the manufacturer’s claim that analysis within 24 h of collection is acceptable. As this is observed for samples with and without thiosulfate, the results further support the conclusion in Section "Sodium thiosulfate does not significantly affect cATP" that addition of sodium thiosulfate is acceptable for cATP analysis.

### Cost and other considerations

While a full cost analysis is beyond the scope of the study, the authors believe that some general remarks on cost comparison between cATP and HPC testing can be valuable to utility managers. As of the date of this writing, the cost of consumables for cATP testing is approximately $20 CAD per sample (LuminUltra® QGA-25), while the cost of a luminometer is approximately $8,000–9,000 CAD (LuminUltra® PhotonMaster). In comparison, HPC costs for utility managers can vary widely depending on the scope of their operations. For utility managers operating certified laboratories for in-house HPC testing (e.g., Metro Vancouver), one of the main consumables is agar growth media. Cost of R2A agar can range from $0.35 CAD per test if prepared from dehydrated powder, up to $17 CAD per test if using prepared plates (Thermo Scientific R2A Agar). This excludes cost of equipment such as incubators, and additional equipment required for media preparation (e.g., drying oven). For utility managers that contract third-party laboratories for HPC testing (e.g., Halifax Water), the cost will naturally vary based on external laboratory pricing. That said, cATP testing is likely to be a cost-effective alternative especially considering its simplicity of operation, with the only equipment required being the aforementioned portable luminometer.

Finally, it is worth noting that a sizeable amount of plastic waste is produced for cATP testing, including the one-time use filters, syringes, and cuvettes. Plastic waste produced in HPC testing varies based on laboratory-specific procedures, with zero waste possible through the use of autoclavable glass plates and pipettes (as is the case for Metro Vancouver). For utility managers using prepared, disposable plates, however, the amount of waste generated becomes comparable with cATP testing.

## Conclusion

This study compares HPC and cATP results from chlorinated distribution system samples from two Canadian utilities in western and eastern Canada, Metro Vancouver (*n* = 40) and Halifax Water (*n* = 283). Samples collected from both systems are characterized by low microbial activity, with median HPC below detection limits of 1–2 CFU/mL. The pristine nature of the studied waters likely contributes to the poor correlation observed between HPC and cATP, with no significant relationship observed for Halifax Water samples, and a moderate correlation observed for Metro Vancouver only when raw source water samples were included. These observations are consistent with the literature on finding poor correlations between HPC and ATP when analyzing water samples with low microbial activity. However, the use of guidelines to interpret HPC and cATP values can result in the same conclusion for more than 95% of the samples, which may suggest a potential framework for using cATP to replace HPC as a faster method for monitoring microbial activity in distribution systems. Additionally, cATP appears to correlate better to free chlorine than HPC as consistent with literature findings, though a significant correlation is observed in only one of the two data sets (Halifax Water). No significant correlations are observed between turbidity and either cATP or HPC.

Two potential operational challenges with implementing cATP assays are its reported incompatibility with chlorine quenching agents like sodium thiosulfate and the time-sensitive nature of cATP. However, this study does not find a statistically significant difference between samples with and without thiosulfate addition (*n* = 15 pairs of data), determining that percent differences between samples with/without thiosulfate are attributable to the coefficient of variance for cATP assays (35 ± 17%, *n* = 26 sets of triplicates). Furthermore, while fluctuations are observed in cATP readings at 4, 6, and 24 h after collection, for both samples with and without added thiosulfate, these percent changes (up to *n* = 13 sample pairs) are not significantly different than baseline variance. As such, the study also supports cATP extraction of samples within 24 h as an acceptable practice. Therefore, the study supports the use of sample bottles pre-charged with sodium thiosulfate (as required for other microbial tests) and lab-extraction (within 24 h) rather than field-extraction of cATP samples for utility monitoring water with low biological activities (e.g., chlorinated water). The advantage of fast turnaround time, together with this flexibility in sample handling procedures, makes ATP testing a viable alternative to HPC for bacteriological monitoring.

## Supplementary Information

Additional file 1. Supplementary Information: Supporting figures and tables.

## Data Availability

The datasets used and/or analysed during the current study are available from the corresponding author on reasonable request.

## Abbreviations

ATP: Adenosine triphosphate
CFU: Colony forming unit
HPC: Heterotrophic plate count
