# Supplementary material for: Evaluating ATP testing for distribution system monitoring: comparison to HPC, impact of chlorine quenching, and hold time dependency
Source: J Biol Eng. 2024 Nov 5;18:63. doi: 10.1186/s13036-024-00446-z (PMC11536932; doi:10.1186/s13036-024-00446-z)
Supplement: Supplementary file 1 — Additional file 1. Supplementary Information: Supporting figures and tables. [file 13036_2024_446_MOESM1_ESM.docx]

##### SUPPLEMENTARY INFORMATION

**Evaluating ATP testing for distribution system monitoring: Comparison to HPC, impact of chlorine quenching, and hold time dependency**

William S. Chen, Leili Abkar, Madjid Mohseni

9 tables

2 figures

Table S1: Historical average HPC and free chlorine of the four sampling sites chosen for triplicate analyses (average period: Jan 1, 2021, to May 30, 2022).

|  | **Low HPC**  *<10 CFU/mL* | **High HPC**  *>60 CFU/mL* |
| --- | --- | --- |
| **Low free chlorine**  *<0.7 mg/L* | **Site 1**  0.62 mg/L  3 CFU/mL | **Site 2**  0 mg/L  520 CFU/mL |
| **High free chlorine**  *>0.7 mg/L* | **Site 3**  1.37 mg/L  5 CFU/mL | **Site 4**  0.76 mg/L  65 CFU/mL |

Table S2: Pearson R coefficients for Halifax Water (n=283) and Metro Vancouver (n=40) samples. Correlations involving free chlorine for Metro Vancouver are limited to n=32 treated water samples as analysis was not performed for n=8 raw inlet samples.

| **Halifax Water** | | | |  | **Metro Vancouver** | | | | | |
| --- | --- | --- | --- | --- | --- | --- | --- | --- | --- | --- |
|  |  |  |  |  |  |  |  |  |  |  |
|  |  | **HPC** | **Free Cl_2_** |  |  |  | **HPC** | **Free Cl_2_** | **Turbidity** |  |
|  |  |  |  |  |  |  |  |  |  |  |
| **cATP** |  | 0.32  (p<0.001) | -0.10  (p=0.1) |  | **cATP** |  | 0.61  (p<0.001) | 0.05  (p=0.8) | 0.08  (p=0.6) |  |
| **HPC** |  |  | 0.12  (p=0.04) |  | **HPC** |  |  | 0.08  (p=0.6) | 0.08  (p=0.6) |  |
|  |  |  |  |  | **Free Cl_2_** |  |  |  | -0.14  (p=0.4) |  |

Table S3: Pearson R (top table) and Spearman Rs (bottom table) coefficients for Metro Vancouver samples excluding raw inlet water samples (n=32). Values for correlations involving free chlorine are the same as those presented in Table S2 (Pearson) and Table 3 (Spearman), as these were already calculated without raw inlet samples.

| **Metro Vancouver** | | | | | | |  |
| --- | --- | --- | --- | --- | --- | --- | --- |
|  |  |  | |  | |  | |
| *Pearson* |  | **HPC** | **Free Cl_2_** | | **Turbidity** | | |
|  |  |  |  | |  | | |
| **cATP** |  | -0.04  (p=0.8) | 0.05  (p=0.8) | | -0.09  (p=0.6) | | |
| **HPC** |  |  | 0.08  (p=0.6) | | 0.20  (p=0.3) | | |
| **Free Cl_2_** |  |  |  | | -0.14  (p=0.4) | | |
|  |  |  |  | |  | | |
| *Spearman* |  | **HPC** | **Free Cl_2_** | | **Turbidity** | | |
|  |  |  |  | |  | | |
| **cATP** |  | -0.02  (p=0.9) | 0.16  (p=0.4) | | 0.04  (p=0.8) | | |
| **HPC** |  |  | 0.27  (p=0.1) | | 0.21  (p=0.3) | | |
| **Free Cl_2_** |  |  |  | | 0.12  (p=0.5) | | |

Table S4: Paired t-test and Wilcoxon signed-rank p-values evaluated for paired cATP results of samples with and without thiosulfate addition, broken down by the time of extraction after sample collection. Shapiro-Wilks test was conducted for differences between paired cATP results (“no thiosulfate” minus “with thiosulfate”) to evaluate normality and, thus, the applicability of paired t-test.

| **Time of extraction** | ***n* data pairs** | **Shapiro-Wilks p-value** | **Paired t-test p-value** | **Wilcoxon signed-rank p-value** |
| --- | --- | --- | --- | --- |
| 4hr | 3 | 0.13 | 0.43 | - |
| 6hr | 6 | 0.03 | - | 0.44 |
| 24hr | 6 | 0.01 | - | 0.84 |

Table S5: Coefficients of variance for cATP, calculated as the standard deviation divided by the mean of triplicate samples. The mean coefficient of variance and one standard deviation are shown, determined from *n* sets of triplicate sample data. One-way ANOVA of all sampling conditions (excluding non-thiosulfate sample extracted 4 hours after collection, which had only n=1 set of triplicates) indicates no significant difference exists between the coefficients of variance (p = 0.4), so the average of all n=26 triplicates (35±17%) was taken as the baseline coefficient of variance of cATP testing.

| **Time of Extraction** | **Coefficient of variance (%)** | |
| --- | --- | --- |
|  | **With Thiosulfate** | **No Thiosulfate** |
| 4hr | 38 ± 15% (n=3) | 33% (n=1) |
| 6hr | 32 ± 13% (n=8) | 24 ± 20% (n=3) |
| 24hr | 45 ± 17% (n=8) | 29 ± 28% (n=3) |

Table S6: To determine the significance of the difference in cATP results with/without sodium thiosulfate, Welch’s t-test p-values are evaluated between cATP percent differences (calculated as “no thiosulfate” minus “with thiosulfate”) versus baseline coefficient of variance of cATP, for three different extraction times (4, 6, and 24 hours after collection).

| **Time of Extraction** | **cATP percent difference** | **Baseline coefficient of variance** | **Welch’s t-test p-value** | |
| --- | --- | --- | --- | --- |
| 4hr | 36 ± 77% (n=3) | 35±17%  (n=26) | | 0.98 |
| 6hr | 18 ± 123% (n=6) |  |  | 0.75 |
| 24hr | 23 ± 89% (n=6) |  |  | 0.76 |

Table S7: Percent change in cATP values between the extraction times studied (i.e., 4, 6, 24 hours) for samples with and without sodium thiosulfate addition. Values shown are mean ± one standard deviation. n is the number of samples.

|  | **Percent Change Between Extractions** | |
| --- | --- | --- |
| **Extraction Time Points** | **With Sodium**  **Thiosulfate** | **Without Sodium Thiosulfate** |
| 4 to 6hr | -75% ± 83% (n=5) | -40% ± 26% (n=3) |
| 6 to 24hr | +19% ± 82% (n=13) | -28% ± 49% (n=7) |
| 4 to 24hr | -30% ± 72% (n=5) | -34% ± 53% (n=3) |

Table S8: (With thiosulfate) To determine the significance of the difference in cATP results when extracted at different time points, Welch’s t-test p-values are evaluated between cATP percent change (absolute value) versus baseline coefficient of variance for cATP. Results are shown for samples with thiosulfate.

| **Extraction Time Points** | **Percent Change** | **Baseline coefficient of variance** | **Welch’s t-test p-value** |
| --- | --- | --- | --- |
| 4 to 6hr | -75% ± 83% | 35±17%  (n=26) | 0.34 |
| 6 to 24hr | +19% ± 82% |  | 0.50 |
| 4 to 24hr | -30% ± 72% |  | 0.88 |

Table S9: (Without thiosulfate) Welch’s t-test values evaluated between cATP percent changes (absolute value) across evaluated time points versus baseline coefficient variance for cATP. Results are shown for samples without thiosulfate addition.

| **Extraction Time Points** | **Percent Change** | **Baseline coefficient of variance** | **Welch’s t-test p-value** |
| --- | --- | --- | --- |
| 4 to 6hr | -40% ± 26% | 35±17%  (n=26) | 0.77 |
| 6 to 24hr | -28% ± 49% |  | 0.72 |
| 4 to 24hr | -34% ± 53% |  | 0.98 |

Figure S1: Residual plot (top left), residual histogram (top right), and Q-Q plot (bottom) for Halifax Water – all samples (n=283)

Figure S2: Residual plot (top left), residual histogram (top right), and Q-Q plot (bottom) for Metro Vancouver – all samples (n=40)
